# Independent Component Analysis-Based Identification of Covariance Patterns of Microstructural White Matter Damage in Alzheimer’s Disease

**DOI:** 10.1371/journal.pone.0119714

**Published:** 2015-03-16

**Authors:** Xin Ouyang, Kewei Chen, Li Yao, Xia Wu, Jiacai Zhang, Ke Li, Zhen Jin, Xiaojuan Guo, for the Alzheimer’s Disease Neuroimaging Initiative

**Affiliations:** 1 College of Information Science and Technology, Beijing Normal University, Beijing, China; 2 Banner Alzheimer’s Institute and Banner Good Samaritan PET Center, Phoenix, Arizona, United States of America; 3 State Key Laboratory of Cognitive Neuroscience and Learning, Beijing Normal University, Beijing, China; 4 Laboratory of Magnetic Resonance Imaging, Beijing 306 Hospital, Beijing, China; Brainnetome Center & National Laboratory of Pattern Recognition, CHINA

## Abstract

The existing DTI studies have suggested that white matter damage constitutes an important part of the neurodegenerative changes in Alzheimer’s disease (AD). The present study aimed to identify the regional covariance patterns of microstructural white matter changes associated with AD. In this study, we applied a multivariate analysis approach, independent component analysis (ICA), to identify covariance patterns of microstructural white matter damage based on fractional anisotropy (FA) skeletonised images from DTI data in 39 AD patients and 41 healthy controls (HCs) from the Alzheimer’s Disease Neuroimaging Initiative database. The multivariate ICA decomposed the subject-dimension concatenated FA data into a mixing coefficient matrix and a source matrix. Twenty-eight independent components (ICs) were extracted, and a two sample *t-test* on each column of the corresponding mixing coefficient matrix revealed significant AD/HC differences in ICA weights for 7 ICs. The covariant FA changes primarily involved the bilateral corona radiata, the superior longitudinal fasciculus, the cingulum, the hippocampal commissure, and the corpus callosum in AD patients compared to HCs. Our findings identified covariant white matter damage associated with AD based on DTI in combination with multivariate ICA, potentially expanding our understanding of the neuropathological mechanisms of AD.

## Introduction

Alzheimer’s disease (AD) is a common progressive neurodegenerative disease among the elderly. The prominent morphological alterations in AD involve not only grey matter atrophy but also white matter damage; for review, see [1,2]. DTI, an advanced non-invasive MRI technique, can quantitatively detect the diffusion characteristics that represent the microstructure of white matter in the brain *in vivo*. The existing DTI studies have suggested that white matter damage constitutes an important part of the neurodegenerative changes associated with AD [3–5]. Multiple DTI indices, such as fractional anisotropy (FA), radial diffusivity (RD), axial diffusivity (AD) and mean diffusivity (MD), can detect the abnormalities in white matter in AD [6–10]. Among these indices, FA, which represents the degree of anisotropy of water diffusion, is one of the most common and important parameters used to characterise the microstructural characteristics of white matter [3,8,11,12].

Earlier DTI studies applied regions of interest (ROI)-based method [13–15]. However, the definition of ROIs requires a priori hypothesis, and this approach is subject to the partial volume effect, especially with respect to small or thin white fibre tracts [3,8]. More recently, some DTI studies performed automated voxel-based style analysis across the entire brain [3,11,16], although misregistration and smoothness-related problems may yield unreliable findings. Then Smith et al. proposed a tract-based spatial statistics (TBSS) method, which utilises intermediate non-linear registration and projection onto the mean FA skeleton image to improve the sensitivity, objectivity and interpretability of voxel-wise analysis of multi-subject DTI studies [17,18].

The above DTI studies applied univariate measures and solely focused on the differences between two groups. However, multivariate approaches, such as independent component analysis (ICA), can elucidate the covariance information hidden in imaging data, revealing the relationships between morphological characteristics and providing a representation of the identified network patterns [19,20]. An ICA model is based on a basic assumption that the unobservable source signals are statistical independent and non-Gaussian. The observed or measured signals, such as brain imaging data, can be considered as linear mixtures of independent components. Therefore, ICA is an unsupervised data-driven statistical method that decomposes linearly mixed signals into maximally independent components carrying similar inter-subject covariance information. Moreover, the mixing coefficients can be used to perform statistical analysis to examine the between-group difference. Determining the alterations in the microstructural connections of white matter associated with AD could play a vital role in understanding the fundamental pathology of AD.

The purpose of the present study was to identify the regional covariance patterns of microstructural white matter changes associated with AD. We performed multivariate ICA on skeletonised FA images from patients with AD and healthy controls (HCs). Moreover, we assessed the discrimination ability between the AD and HC groups using the subject-specific mixing coefficients for each independent component.

## Materials and Methods

Data used in the preparation of this article were obtained from the Alzheimer’s Disease Neuroimaging Initiative (ADNI) database (adni.loni.usc.edu). The ADNI was launched in 2003 by the National Institute on Aging (NIA), the National Institute of Biomedical Imaging and Bioengineering (NIBIB), the Food and Drug Administration (FDA), private pharmaceutical companies and non-profit organizations, as a $60 million, 5-year public-private partnership. The primary goal of ADNI has been to test whether serial magnetic resonance imaging (MRI), positron emission tomography (PET), other biological markers, and clinical and neuropsychological assessment can be combined to measure the progression of mild cognitive impairment (MCI) and early Alzheimer’s disease (AD). Determination of sensitive and specific markers of very early AD progression is intended to aid researchers and clinicians to develop new treatments and monitor their effectiveness, as well as lessen the time and cost of clinical trials.

The Principal Investigator of this initiative is Michael W. Weiner, MD, VA Medical Center and University of California—San Francisco. ADNI is the result of efforts of many co-investigators from a broad range of academic institutions and private corporations, and subjects have been recruited from over 50 sites across the U.S. and Canada. The initial goal of ADNI was to recruit 800 subjects but ADNI has been followed by ADNI-GO and ADNI-2. To date these three protocols have recruited over 1500 adults, ages 55 to 90, to participate in the research, consisting of cognitively normal older individuals, people with early or late MCI, and people with early AD. The follow up duration of each group is specified in the protocols for ADNI-1, ADNI-2 and ADNI-GO. Subjects originally recruited for ADNI-1 and ADNI-GO had the option to be followed in ADNI-2. For up-to-date information, see www.adni-info.org/.

### Ethics statement

The Alzheimer’s Disease Neuroimaging Initiative (ADNI) study was approved by Institutional Review Board (IRB) of each participating site including University of Southern California and Banner Alzheimer’s Institute and was conducted in accordance with federal regulations and the Internal Conference on Harmonisation (ICH) guidelines of Good Clinical Practice (GCP). The study subjects provided their written informed consent at the time of enrolment regarding imaging data and completed questionnaires approved by each participating site’s IRB.

### Subjects

According to the ADNI protocol, the diagnosis of probable AD met the National Institute of Neurological and Communicative Disorders and Stroke/Alzheimer’s Disease and Related Disorders Association (NINCDS/ADRDA) criteria, and the severity of cognitive impairment was determined based on the Mini-Mental State Examination (MMSE) and Clinical Dementia Rating (CDR) scores. In this investigation, the participants included 39 AD patients (23 males and 16 females, mean age: 74.91±8.13 years, range: 60–90 years; mean Mini-Mental State Examination (MMSE) score: 22.87±2.32, range: 18–27; CDR score: 0.5 or 1) and 41 HCs (20 males and 21 females, mean age: 73.97±6.34 years, range: 60–90 years; MMSE score: 29.07±0.96, range: 27–30; CDR: 0). The AD group did not significantly differ from the HC group with respect to gender ratio ($\chi_{\left(1\right)}^{2}=0.836,p=0.361$) or age (*t*_(78)_ = 0.575,*p* = 0.567) but exhibited significantly lower MMSE scores (*t*_(78)_ = -15.768,*p* = 5.693e-026).

### DTI data acquisition

All DTI scans were acquired using 3T GE MEDICAL SYSTEM scanners at the various ADNI sites. All scans were collected using the standard ADNI MRI protocol. For each subject, the scans were collected using the following parameters: pulse sequence = EP/SE; matrix size = 256 mm × 256 mm; flip angle = 90°; slice thickness = 2.7 mm; gradient directions = 41 (b = 1000 s/mm^2^) and 5 acquisitions without diffusion weighting (b = 0 s/mm^2^).

### DTI data pre-processing

The DTI data were pre-processed using the FMRIB Software Library (FSL) version 5.0 (http://www.fmrib.ox.ac.uk/fsl). Using FMRIB’s Diffusion Toolbox (FDT) version 2.0, the eddy current correction and the head motion correction were applied via affine registration of each subject’s diffusion-weighted image to the non-diffusion-weighted image. The non-brain structures were removed using the Brain Extraction Tool, and the FA maps were generated based on the diffusion tensor reconstructed using the DTIfit program. Then, all subjects’ FA images were processed using TBSS analysis [17,18]: first, each subject’s FA image was nonlinear registrated to the MNI space; second, the mean FA image was calculated and thinned to generate the mean FA skeleton image (FA > 0.2), which represents the centre of white matter tract; third, each subject’s transformed FA image was projected to the mean FA skeleton image by calculating the maximum FA values from the nearest tract centre and filling the corresponding position in the skeleton. Lastly, all subjects’ skeletonised FA images were utilised for multivariate ICA.

### Multivariate ICA

ICA was performed using the Fusion ICA toolbox (FIT) version 2.0c (http://icatb.sourceforge.net/). FIT toolbox is used to examine the shared information between the features and it can also be used to analyze one modality data. In the current study, we did not apply fusion theory and just analyzed DTI data using single modality ICA.

The ICA model can be written as

X=AS,

where X is an observed data matrix, A is an unknown mixing matrix and S is a source matrix that refers to the statistically independent source signals. The ICA model assumes that X is generated by linearly mixing A and S.

In this study, the skeletonised FA image of each subject was arrayed into a one-dimensional vector, and the input data matrix X (size: subject number by voxel number) was generated by concatenating the skeletonised FA data (a row vector) of all subjects from the two groups, AD patients and HCs. The initial data matrix X was decomposed using ICA based on the Infomax algorithm to obtain the mixing coefficient matrix A (size: subject number by source number) and the source matrix S (size: source number by voxel number) [21]. Each column of the mixing matrix A (ICA weights) indicates the degree to which each subject expresses the source or network differences between two groups. Each row of the source matrix S represents the source maps exhibiting the inter-subject covariance information.

### Statistical analysis

A two-sample *t*-test was performed on each column of the mixing coefficient matrix (ICA weights) to examine the between-group differences (p<0.05, false discovery rate (FDR) corrected for multiple comparisons). Each significant source (the row of source matrix) was converted to zero mean and unit standard deviation (Z-scores), and then transferred to a three-dimensional brain map with a threshold of Z ≥3 to reflect the significant white matter covariant pattern.

Receiver operating curve (ROC) analysis was implemented to assess the discrimination ability of the mixing coefficients for each IC. The sensitivity is defined as a/(a+c) in which a is the number of AD subjects that are correctly identified and c is the number of AD subjects that are not correctly identified. The specificity is defined as d/(b+d), in which d is the number of HCs that are correctly identified and b is the number of HCs that are not correctly identified. Besides, a multivariate ROC (multiV-ROC) was applied to combine the ICA weights (i.e., the column of the mixing matrix which showed significant between-group differences in the two-sample *t*-test) [22].

## Results

Twenty-eight ICs were extracted, of which ICA weights of 7 ICs displayed significant differences between the AD and HC groups. Table 1 presents the locations of the predominant FA changes in each IC spatial map of AD patients compared to HCs.

**Table 1:** Locations of the covariant FA changes for each IC spatial map in AD patients compared to HCs

	Independent Components	Primary White Matter Regions
**Projection fibres**	**IC 1**	Left and right anterior corona radiata Left and right superior corona radiata
	**IC 2**	Left and right posterior thalamic radiation (including the optic radiation)
	**IC 3**	Left and right retrolenticular part of the internal capsule
**Association fibres**	**IC 4**	Left and right superior longitudinal fasciculus
	**IC 5**	Left and right cingulum
**Commissural fibres**	**IC 6**	Hippocampal commissure
	**IC 7**	Genu and anterior body of the corpus callosum

**Fig 1:**
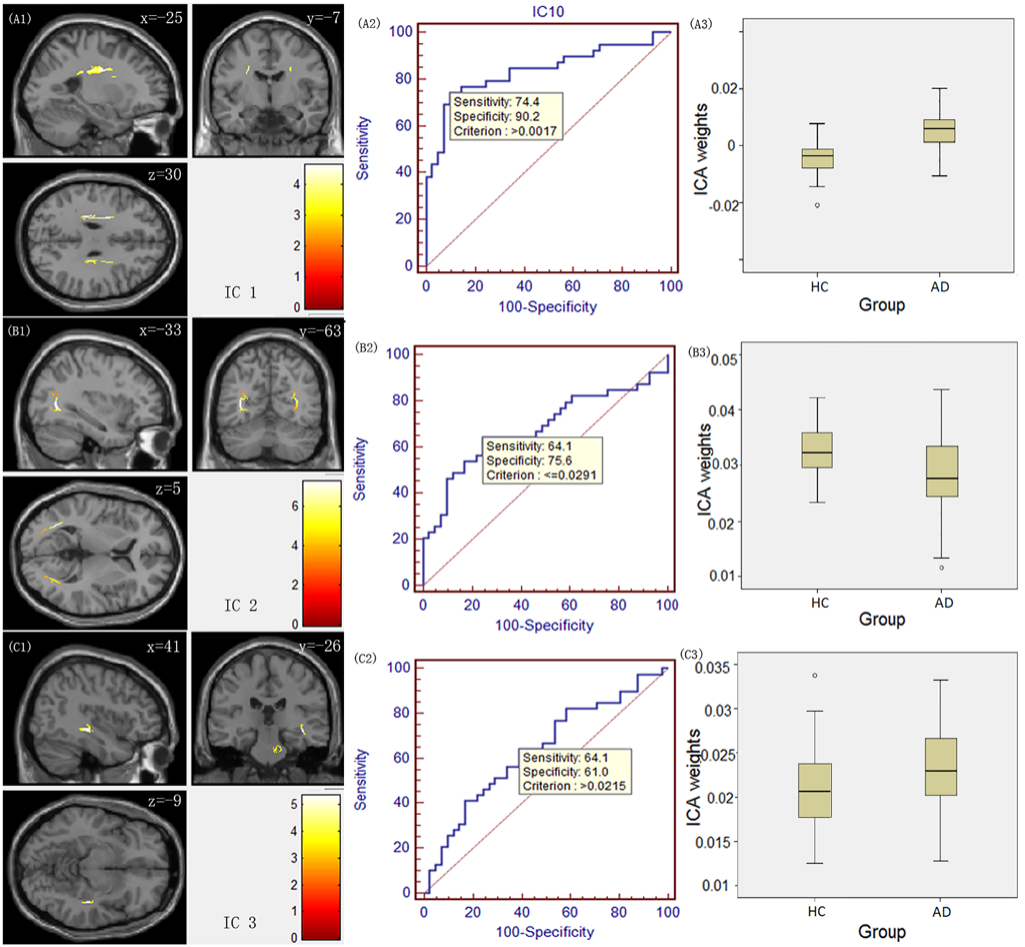
IC spatial maps displaying the covariant FA changes in white matter in AD patients compared to HCs for ICs 1–3. The colour bar represents Z-score. The middle panel displays their corresponding receiver operating characteristic (ROC) curves, and the right panel displays a boxplot of the between-group differences in ICA weights.

**Fig 2:**
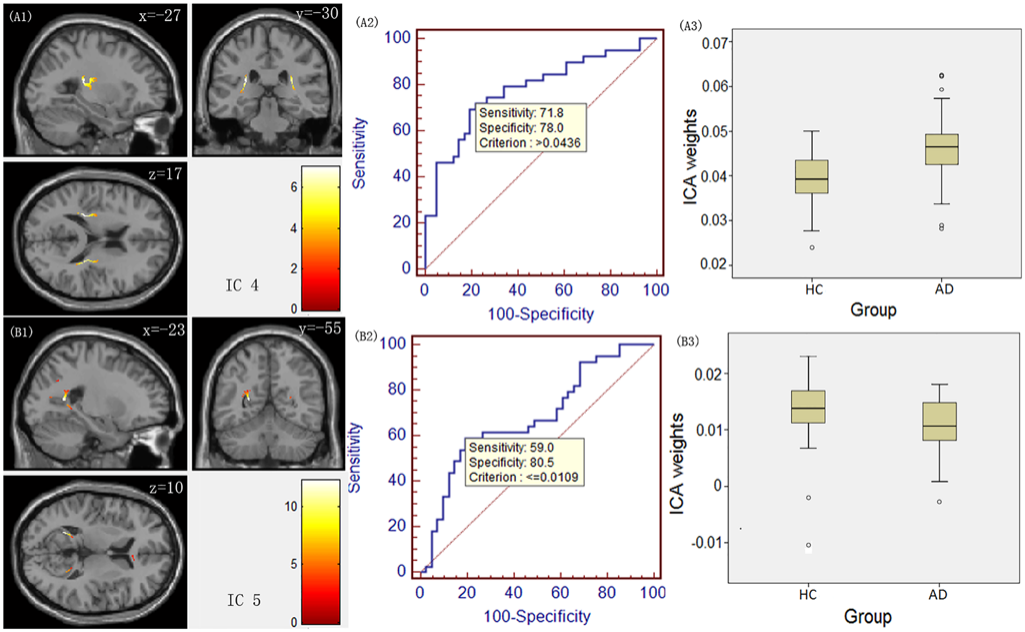
IC spatial maps displaying the covariant FA changes in white matter in AD patients compared to HCs for ICs 4 and 5. The colour bar represents Z-score. The middle panel displays their corresponding receiver operating characteristic (ROC) curves, and the right panel displays a boxplot of the between-group differences in ICA weights.

**Fig 3:**
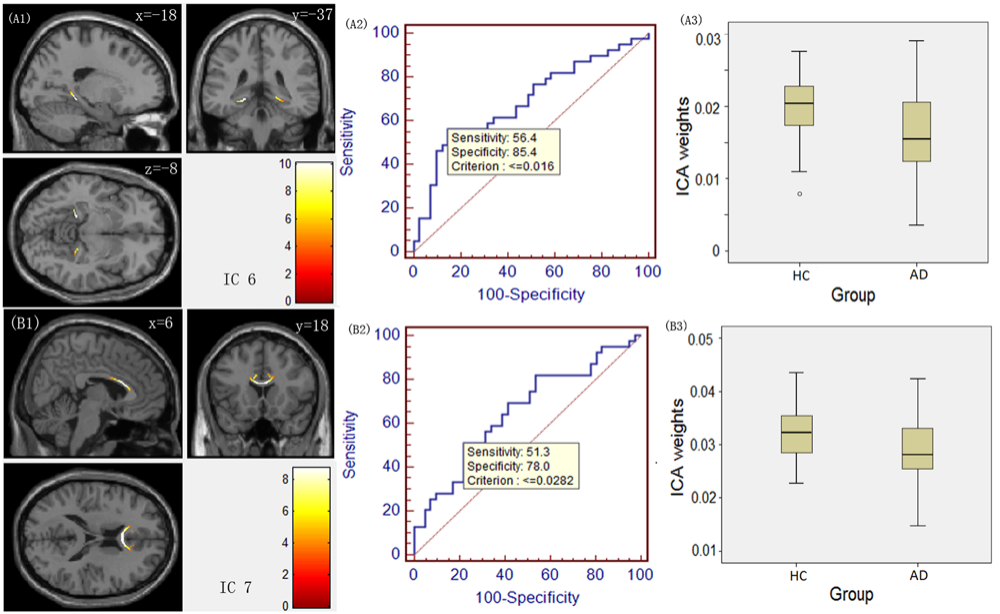
IC spatial maps displaying the covariant FA changes in white matter in AD patients compared to HCs for ICs 6 and 7. The colour bar represents Z-score. The middle panel displays their corresponding receiver operating characteristic (ROC) curve, and the right panel displays a boxplot of the between-group differences in ICA weights.

Figs. 1–3 illustrate the spatial maps of the covariant changes in the FA value in AD patients compared to HCs (Fig. 1 (A1, B1, C1), Fig. 2(A1, B1), Fig. 3(A1, B1)) for 7 ICs, and the right panels display their corresponding ROC curves (Fig. 1 (A2, B2, C2), Fig. 2(A2, B2), Fig. 3(A2, B2)) and the boxplot of the between-group differences in their ICA weights (Fig. 1 (A3, B3, C3), Fig. 2 (A3, B3), Fig. 3 (A3, B3)). The positively weighted coefficients in the IC spatial maps displayed the covariant changes in the FA value in AD patients compared to HCs. In addition, the IC spatial maps with negatively weighted value (with a threshold of Z ≤ -3) are presented in S1 Fig.

For IC 1, the source primarily consisted of the bilateral anterior and superior corona radiata (Fig. 1 (A1)). IC 2 included the bilateral posterior thalamic radiation (Fig. 1 (B1)), and IC 3 (Fig. 1 (C1)) consisted primarily of the bilateral retrolenticular part of the internal capsule. The above three ICs represent components of the projection fibres. As for the association fibres IC 4 and IC 5, they primarily involved the bilateral superior longitudinal fasciculus (Fig. 2 (A1)) and the bilateral cingulum (Fig. 2 (B1)), respectively. IC 6 predominantly encompassed the hippocampal commissure (Fig. 3 (A1)), and IC 7 included the genu and the anterior body of the corpus callosum (Fig. 3 (B1)), which are identified as components of the commissural fibres.

Regarding the between-group differences and the ROC results measured by ICA weights, ICA weights for IC 1, IC 4 and IC 6 had the highest statistical power (t_(78)_ = 6.272, p = 1.85E-8 for IC 1; t_(78)_ = 4.592, p = 1.66E-5 for IC 4; and t_(78)_ = -3.021, p = 0.0034 for IC 6). The ROC analysis revealed discrimination ability with 74.4%, 71.8% and 56.4% sensitivity for IC 1, 4 and 6, respectively; 90.2%, 78.0% and 85.4% specificity for IC 1, 4 and 6, respectively. In addition, the result of multiV-ROC revealed the discrimination ability with 89.7% sensitivity and 90.2% specificity.

## Discussion

In the present study, we performed ICA, a multivariate analysis method, on DTI data from both AD patients and HCs to identify covariance patterns of microstructural white matter damage associated with AD. Twenty-eight ICs were extracted and two-sample *t*-test on the ICA weights for seven ICs revealed that the between-group differences were significant in AD patients compared to HCs.

For IC 1 to IC 3, the covariant FA changes primarily consisted the projection fibres, including the anterior and superior corona radiata, the posterior thalamic radiation and the retrolenticular part of the internal capsule, generally corresponding with previous studies [8–10,12,14]. Kincses et al. performed linked ICA analysis on four DTI indices to investigate the pattern of changes in diffusion parameters in AD and found that the decreased FA primarily involved the forceps major, the corona radiata and the superior longitudinal fasciculus, partially in accordance with our results [9]. In contrast to the study by Kincses et al., our analyses detected more significant FA abnormalities in more ICs of white matter regions.

For IC 4 and IC 5, their corresponding between-group differences in AD and HCs were significant and the covariant FA changes were found in the association fibres, especially the superior longitudinal fasciculus and the cingulum, in accordance with previous studies [8,9,12,14]. The superior longitudinal fasciculus connects the frontal, parietal, occipital and temporal lobes, and the fibres in the cingulum connect the posterior cingulate cortex and the medial prefrontal cortex. Guo et al. performed joint ICA on grey and white matter volume maps to construct the covariant networks and found grey matter atrophy in the middle/inferior/superior frontal and cingulate gyri, the hippocampus and the parahippocampal gyrus and joint reductions in white matter volume in the related superior longitudinal fasciculus, the corpus callosum and the corona radiata [23]. These results suggest that the underlying white matter connectivity may be associated with grey matter atrophy in these brain areas. The damage to the cingulum fibres may reflect axonal loss or demyelination that disconnects the posterior cingulate gyrus from the hippocampus [24]. Moreover, the medial temporal lobe and the posterior cingulate gyrus are essential to memory function [24,25]. These findings suggest that these association fibres are associated with the memory deficit in AD.

FA value varies from zero to one and describes the degree of diffusion anisotropy. Zero means that diffusion is equally restricted in all directions while one means diffusion is along only one direction. Generally, decreased FA value is caused by a breakdown in white matter fibers. However, fibers in crossing pathways have more than one direction and damaged white matter in such regions may lead to reduced number of directions thus to higher FA values. It is worth noting that, in our study, ICA weights for IC 1, IC 3 and IC 4 were higher in AD compared to HCs. While it is possible that the elevated FA in IC1, IC3 and IC4 represents the effect of reduced fiber crossing owing to degeneration of crossing pathways in AD, we note the individual source map represents the contributions of the corresponding FA to the overall connected system. They themselves are not FA values. Regardless the possible cause of the observed higher ICA weights in AD patients, our findings should be further investigated with additional studies designed to address the causes behind.

For IC 6 and IC 7, the majority of the reductions in the FA value in AD patients were detected in the hippocampal commissure and the genu and the anterior body of the corpus callosum, components of the commissural fibres; these results were similar to those of other studies [8,13,14,23,26]. Previous studies demonstrated that the reduction in grey matter volume in the hippocampus was a valuable biomarker for diagnosis of AD [23]. Therefore, the hippocampal commissure damage in this study was related to alterations in the hippocampus. In addition, the genu, body, and splenium of the corpus callosum were detected as important tracts connecting two resting-state networks, the bilateral prefrontal cortical regions and posterior precuneus regions [27]. Frisoni et al. reported reductions in grey matter density involving the frontal cortex and the precuneus, which indicates that disconnecting white matter fibres in the corpus callosum may influence the above two grey matter regions and the function of resting-state networks in AD [28].

The default mode network (DMN), an important resting-state network, has been generally studied, and it has been found to be altered in AD [29,30]. Previous studies indicated that understanding structural connectivity would facilitate the understanding of functional connectivity [29,31]. The superior longitudinal fasciculus, the cingulum and the corpus callosum are essential for connecting the regions involved in the DMN [27,32]. Li Luo et al. also found that anterior corona radiata and the anterior limb of the internal capsule were strongly associated with the DMN [32]. Those tracts are similar to our findings, which may definitively demonstrate that alterations in the projection fibres may reflect a disconnection of this functional network in AD. Greicius et al. found that AD patients exhibited decreased resting-state activity in the posterior cingulate and the hippocampus, regions which play important roles in the DMN [29]. We hypothesise that the disconnection of the above-mentioned fibres actually affects the activity of the DMN.

As for the ROC results and the between-group differences in the ICA weights, ICA weights for IC 1, IC 4 and IC 6 displayed the highest statistical power for between-group significance. Each IC may reflect a different aspect of the pathological changes in AD, and these ICs may represent valuable AD pathology biomarkers for prediction and diagnosis. In addition, the result of multiV-ROC revealed the discrimination ability with 89.7% sensitivity and 90.2% specificity and suggested the increased statistical power of distinguishing AD patients from HCs when using the combined ICA weights than each ICA separately.

Unlike univariate analysis methods (ROI, VBA, TBSS) which can only examine voxel by voxel alterations, the multivariate ICA approach, without any priori knowledge, can successfully identify the regional covariance patterns of microstructural white matter abnormalities associated with AD. In brief, compared with univariate analysis methods, ICA has three advantages, first, ICA decomposes the skeletonised FA maps into ICs and voxels of each IC carry covariance information. Second, the mixing coefficients can clearly explain the subjects’ contribution to the corresponding source. Third, ICA establishes a global index (subject score) free of multiple comparisons typically associated with univariate approaches. Other diffusion tensor measures (such as RD, AD, and MD) can provide different information to explain the nature of white matter changes and could be examined using ICA in future studies.

However, in this study, the multivariate ICA was applied on the subject-dimension concatenated data from AD patients and HCs together. Such performance, based on the assumption that subjects from the two groups have common ICs, could be biased. Such approach has been reportedly used in some previous studies [33–35]. With this approach, the resultant ICs should be interpreted with caution as the characteristics over which the group differences can be expressed. Owing to the direction of ICs is arbitrary mathematically, we also examined the negatively weighted values of the ICs (S1 Fig.). There were few significant findings in spatial source maps for IC 1 and ICs 3–7 and no any findings for IC 2. However, we noticed that the positively weighted results reported in this study are primarily consistent with the existing literature. Another limitation of this study is we did not test the discrimination ability using new independent dataset from AD patients and HCs. All the ROC curves were obtained based on the original data. Besides, mild cognitive impairment (MCI) is a transitional stage between normal aging and AD and it is valuable to apply the unearthed common ICs to MCI to see their predictability for the progression to AD.

In summary, we used ICA to identify the microstructural white matter abnormalities associated with AD, and these findings may expand our understanding of the neuropathological mechanisms of AD at a network level. In addition, multimodal fusion is an effective method to better investigate brain networks. In a further study, we shall perform multivariate methods, such as joint ICA, on both structural MRI and DTI data for joint analysis to comprehensively investigate the anatomical changes in AD.

## Supporting Information

S1 Fig The IC spatial maps with negatively weighted value (with a threshold of Z ≤ -3) for IC 1 and ICs 3–7. The colour bar represents Z-score. Notice that IC 2 has no significant negative result and we did not include it in this figure.
(TIF)
