# Supplementary figures and images for: Independent Component Analysis-Based Identification of Covariance Patterns of Microstructural White Matter Damage in Alzheimer’s Disease
Source: PLoS One. 2015 Mar 16;10(3):e0119714. doi: 10.1371/journal.pone.0119714 (PMC4361402; doi:10.1371/journal.pone.0119714)

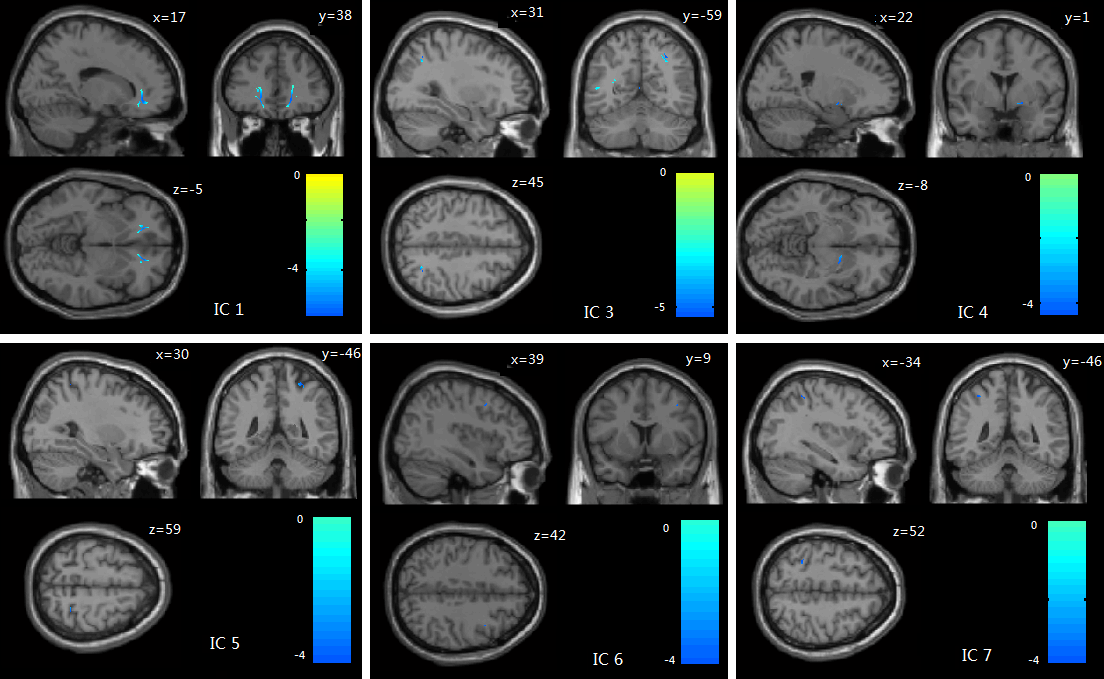

Supplement: S1 Fig — The colour bar represents Z-score. Notice that IC 2 has no significant negative result and we did not include it in this figure. (TIF) [file pone.0119714.s001.tif]
